# GABAergic neuroactive steroids: a new frontier in bipolar disorders?

**DOI:** 10.1186/1744-9081-8-61

**Published:** 2012-12-19

**Authors:** Mauro Giovanni Carta, Krishna M Bhat, Antonio Preti

**Affiliations:** 1 Department of Public Health, Clinical and Molecular Medicine, University of Cagliari and Center for Consultation-Liaison Psychiatry and Psychosomatics University Hospital of Cagliari, Cagliari, Italy; 2 Department of Neuroscience and Cell Biology, University of Texas Medical Branch, Galveston, Texas, USA

## Abstract

Neurosteroids are synthesized in the brain and modulate brain excitability. There is increasing evidence of their sedative, anesthetic and antiseizure properties, as well as their influence on mood. Currently neurosteroids are classified as pregnane neurosteroids (allopregnanolone and allotetrahydrodeoxycorticosterone), androstane neurosteroids (androstanediol and etiocholanone) or sulfated neurosteroids (pregnenolone sulfate and dehydroepiandrosterone sulfate). Both preclinical and clinical findings indicate that progesterone derivative neurosteroids such as allopregnanolone and allotetrahydrodeoxycorticosterone play a role in mood disorders. Clozapine and olanzapine, which were shown to be effective in stabilizing bipolar disorder, elevate pregnenolone levels in rat hippocampus, cerebral cortex, and serum. In lithium-treated mice, the blood levels of allopregnanolone and pregnenolone were elevated compared to control levels. Women diagnosed with bipolar disorder typically show symptomatic exacerbation in relation to the menstrual cycle, and show vulnerability to the onset or recurrence of mood disorders immediately after giving birth, when the levels of neurosteroid derivatives of progesterone drop. Whereas in women who had recovered from bipolar disorder, the plasma concentration of allopregnanolone was elevated compared to either healthy controls or women with major depressive disorder during the premenstrual period. During depressive episodes, blood level of allopregnanolone is low. Treatment with fluoxetine tends to stabilize the levels of neurosteroids in depression. These findings converge to suggest that these steroids have significant mood-stabilizing effect. This hypothesis is consistent with the observation that a number of anticonvulsants are effective therapies for bipolar disorder, a finding also consistent with the antiseizure properties of neurosteroids. Further exploration of action of neuroactive steroids is likely to open new frontiers in the investigation of the etiology and treatment of mood disorders, particularly bipolar disorders.

## Introduction

Pharmacotherapy of severe mental disorders has not changed significantly since the introduction of antipsychotic compounds in the 1950s. The current classes of drugs available to treat schizophrenia, bipolar disorder and major depression involve essentially the same mechanisms of action and the same neurobiological target [1]. Arguably the most important advance in the pharmacotherapy of severe mental disorders in the last fifty years was the substitution of barbiturates with the clinically safer benzodiazepines and the introduction of the theory-driven selective serotonin reuptake inhibitors for the treatment of depression. Nevertheless, the effectiveness of currently available drugs is poor, both because only selected subgroups of patients were found to be good responders to the prescribed drugs [2,3] and because compliance to treatment is generally low because of side effects and poor insight on the necessity of a severe mental disorder needing long-term therapy [4,5].

The development of new therapeutic targets in the treatment of severe mental disorders is hampered by the lack of external, biological markers of the nosographic phenotypes [6,7], the scarce knowledge of the neurobiological and genetic substrates of the categorically defined disorders [8] and the difficulties in devising valid and reliable animal models, which often lack predictive validity in the prediction of drug actions in humans [9]. Therefore, serendipity is as likely to guide discovery now as it was in the past. However, it is always possible to help serendipity-assisted drug discovery by looking at the crossroads of epidemiology with laboratory and clinical research.

### Gender issues in the epidemiology of severe mental disorders

There is robust epidemiological evidence that patients diagnosed with schizophrenia and mood disorders show gender differences in the onset, course and outcome of their disorders. Since the German ABC study (Age, Beginning, Course) on schizophrenia, it is well known that females have two peaks in the onset of schizophrenia, contrary to males [10,11]. Recent systematic reviews and meta-analyses confirmed that males are over-represented in the samples of patients diagnosed with first-episode schizophrenia. They are generally younger at first contact, and tend to have a poorer outcome compared to females [12-15]. To account for these findings, it was suggested that estrogens may have modulated dopaminergic hyperactivity in females, thus leading to a gradual progression of the course of the disorder and a later onset of frank psychosis [11,16,17]. This hypothesis was compatible with the two incidence peaks in female psychosis onset; one in the early 20s, also common among males, albeit slightly later, and another after 40 years, possibly related to menopause [10,18].

Mood disorders show a different picture, with women having an increased risk for developing major depression compared to males [19]. The risk of hypomania, rapid cycling and mixed episodes is also higher among women than men [20]. However, men and women suffer the same incidence of bipolar disorder and essentially with the same outcome [19,20]. If steroids have a role in schizophrenia related psychoses, this role is reversed in affective psychoses. The discovery that the brain can synthesize neuroactive steroids and that their action is widespread on neuronal cells opened up an entirely new area in the investigation on rge effects of steroids on behavior [21,22].

### Sex steroids and the brain

Peripherally, steroids are produced mainly by the adrenal cortex and the gonads and are regulated by the hypothalamic-pituitary-adrenal axis and the hypothalamic pituitary-adrenal gonadal axis through negative feedback. The ovarian steroids regulate neuroendocrine, endocrine and behavioral functions through a number of cellular mechanisms. Typically, both estrogen and progesterone induce a relatively long-term action on neurons by activating a number of intracellular receptors that modulate the transcription and protein synthesis.

The steroid hormones play an important role in both the central and peripheral nervous systems; they act during development, growth, maturation and cellular differentiation. The progesterone receptor and the nuclear hormone family of intracellular estrogen receptors, alpha and beta (encoded by two separate genes), are dimeric molecules that regulate transcription of target genes in the nuclei [23,24]. These receptors have a C-terminal and an N-terminal zinc-finger domains that mediate binding of the receptors to target DNA sequences. In the absence of the binding hormone, the C-terminal of the receptors inhibits transcription. The binding of the hormone induces a change in the dimeric structure of the receptors, and this removes the inhibitory action. Estrogens also bind to a G-protein coupled receptor, which as a transmembrane G-protein mediates estrogen-dependent kinase activation [25].

Both estradiol and progesterone receptors are found in the Central Nervous System (CNS) [26]. Progesterone is rapidly absorbed and metabolized in the cerebral cortex [27]. Because of their lipophilic nature, the steroids produced by the endocrine glands pass freely through the blood–brain barrier. The concentration of estradiol and progesterone in the brain closely follows peripheral concentrations. The brain is one of the targets of steroid hormones [28,29]. In general, estradiol induces excitatory actions while progesterone induces inhibitory actions on the CNS. The ovarian steroids modulate many functions of the CNS, such as memory and learning [30], movement [31], and the perception of pain [32]. The ovarian steroids regulate neuroendocrine, endocrine and behavioral functions through a number of cellular mechanisms.

### Neurosteroids and their role in the brain

Baulieu and co-workers were the first to observe that the brain concentrations of dehydroepiandrosterone sulfate were partially independent from adrenal and gonad secretion [33]. Subsequently, the demonstration of de novo synthesis of active steroids in the brain leads to the conceptualization of these brain-acting steroids as neurosteroids [21]; neurosteroids are synthesized from circulating steroid hormones, which serve as precursors of active neurosteroids. Currently, neurosteroids are defined as those that are synthesized in the brain. Neuroactive steroids refer to steroids that, independent of their origin, are capable of modifying neural activities.

Currently neuroactive steroids are classified as pregnane neurosteroids (allopregnanolone and allotetrahydrodeoxycorticosterone or THDOC), androstane neurosteroids (androstanediol and etiocholanone) or sulfated neurosteroids (pregnenolone sulfate or PS and dehydroepiandrosterone sulfate or DHEAS).

Several enzymes are involved in the synthesis of neuroactive steroids: neuroactive steroids such as allopregnanolone, THDOC, and androstanediol are produced by 5α-reductase and 3α-hydroxysteroid oxidoreductase (3α-HSOR), which act by reducing the parent steroid in peripheral tissues, such as liver and skin [34]. Both the 5α-reductase and the 3α-HSOR were identified in both neural and glial cells [35,36] and found in neocortex and subcortical white matter and in hippocampal tissues [37,38]. In particular, a cytochrome P450 cholesterol side-chain cleavage enzyme (CYP450scc) was identified, and was proven to have the ability to convert cholesterol to pregnenolone, which is a precursor for the synthesis of neurosteroids [39]. Another enzyme necessary for the conversion of pregnenolone to progesterone, 3β-hykdroxysteroid dehydrogenase, was found in the brain [40]; further details on the synthesis of neuroactive steroids in [41].

In the brain de novo synthesis of neurosteroids occurs in the cortex, the hippocampus and the amygdala, mainly in glutamatergic neurons [27]. Regulatory mechanisms involved in this de novo synthesis are still unclear [42].

### Non-genomic actions of neurosteroids

There is evidence that neurosteroids do not produce most of their effects through an interaction with the steroid hormone receptors that regulate gene transcription. But, neurosteroids can regulate gene expression via the progesterone receptor. But this occurs only after conversion of the neurosteroids to typical steroids. The induction of DNA binding and transcriptional activation of the progesterone receptor requires intracellular oxidation of the neuroactive steroids into progesterone receptor active 5 alpha-pregnane steroids [43]. Most effects of neurosteroids occur by interaction with neuronal membrane receptors and ion channels [44]. The post-synaptic GABA_A_ receptor is the most important site where neuroactive steroids act as positive or negative regulators [19], which is consistent with their chemical structure.

### Neurosteroids involvement in the regulation of GABAergic transmission

The GABA_A_ receptor is the main target of action of neuroactive steroids. GABA_A_ receptors are heteropentameric GABA-gated chloride channels: they are involved in fast inhibitory neurotransmission. The GABA_A_ receptor, distributed in large quantity throughout the CNS, is a macromolecular complex consisting of five subunits, of which many homologs have been identified (alpha1-6, beta1-3, gamma1-3, delta, epsilon, theta, pi and rho1-3). In each receptor, these five subunits are assembled from among 19 different subunit isoforms [45]. Composition of the five subunits determines the functional and pharmacological properties of GABA_A_ receptors. The best-known and most widely distributed form in the CNS consists of two alphas, two betas, and a third subunit, which together constitute the chloride ion channel. Different assemblages of the five subunits determine differences in the functioning of the channel and differences in the response to the drugs acting on the receptor [46].

Site-specific binding of the GABA_A_ receptor, in subunit beta, determines the opening of the ion channel and chloride influx. The increase in negative charge leads to a hyperpolarization of the membrane, making it less susceptible to excitation [46]. The activation of GABA_A_ receptors prevents a potential short circuit in the depolarization induced by excitatory neurotransmitters. The activity of these receptors is also modulated by a number of agents including benzodiazepines, barbiturates, some anesthetics and ethanol [47].

The neurosteroids allopregnanolone and THDOC are strong positive allosteric modulators of the GABA_A_ receptors [48], but at a different site than the site bound by the barbiturates and the benzodiazepines [48]. The neuroactive steroids increase the flow of chloride ions from GABA_A_ receptors, by increasing both the frequency and duration of the opening of the ion channel [49]. Due to the increased probability of opening of the chloride channel of the GABA_A_ receptor, neuroactive steroids increase a massive influx of the ion and potentiate inhibitory GABAergic transmission [49].

In vivo, the basal plasma concentration of *neuroactive steroids* seems sufficient to significantly potentiate the function of the GABA_A_ receptor [50]. Neuroactive steroids can also modulate recombination of various subunits of the GABA_A_ receptor [51]. This recombination can change the action of substances such as alcohol and drugs on the GABA_A_ receptor: the action of these substances on the GABA_A_ receptor depends on receptor assembly [52].

The steroid sulfates are non-competitive antagonists of the GABA_A_ receptors, acting on different sites from those bound by allopregnanolone and THDOC [53]. The negative modulatory action of neuroactive steroids is produced through a reduction in channel opening, but the precise mechanism of this blocking is still not well understood [54]. The neuroactive steroid sulfates also modulate GABAergic transmission through a poorly known presynaptic mechanism [55]. Given the abundance of pregnenolone sulfate (PS) and dehydroepiandrosterone-3-sulfate (DHEAS) in the brain, it seems likely that they can act as endogenous neuromodulators [56]; this in conjunction with neurosteroids such as allopregnanolone and THDOC, which are strong positive allosteric modulators of the GABA_A_ receptors.

Studies carried out in amphibians showed that GABA, through its GABA_A_ receptors, is involved in the control of neurosteroidogenesis, with GABA inhibiting it [56]. Therefore, a short, regulatory loop linking neuroactive steroids’ effects to GABA actions might exist.

### The study of progesterone derivatives in epilepsy and mood disorders

The role of allopregnanolone and THDOC has been studied in a number of pathologies including epilepsy [57], premenstrual syndrome [57], anxiety [58] and mood disorders [59,60].

These steroids show a protective action in epilepsy and elevate the threshold of convulsive crisis, particularly in so-called catamenial epilepsy, in which the crises worsen during the premenstrual phase. Indeed, clinical studies have demonstrated the potential therapeutic utility of synthetic analogs of allopregnanolone in the treatment of catamenial epilepsy [57]. The reduction in the concentration of progesterone derivatives in the luteinizing phase of the menstrual cycle impacts on the clinical manifestations of premenstrual syndrome and premenstrual dysphoric disorder (PMDD) [61], and it is consistent with these findings. PMDD is associated with mood disorders [62], and during depressive episodes, the level of allopregnanolone is low [63]. Conversely, the plasma concentration of allopregnanolone is elevated in patients with panic disorder [64], or following a panic attack [65].

The treatment with fluoxetine stabilizes the level of neurosteroids in depression and panic, and it has been hypothesized that at least part of the therapeutic effect of selective serotonin re-uptake inhibitors (SSRIs) could be through their influence on neurosteroids [66,67]. Recent findings indicate that neurosteroids such as dehydroepiandrosterone, pregnenolone and their sulfate esters (progesterone and allopregnanolone) affect neuronal survival, neurite outgrowth and neurogenesis [68]. Re-establishment of neuronal plasticity (dendritic remodeling and synaptic contacts) in the hippocampus may be important for the pathogenesis and amelioration of depressive symptoms [69]. Neurosteroids might have a role in resetting neurogenesis in some areas of the brain, and specifically in the hippocampus during recovery from depressive episodes.

### A role for progesterone-derived neurosteroids in bipolar disorders?

In women with bipolar disorders during euthymia, plasma concentration of the progesterone derivative allopregnanolone is elevated in the premenstrual period compared to healthy controls and women with major depressive disorder [70]. This finding is independent from pharmacological therapy status and not related to anxiety or eating disorders. It was speculated that these neurosteroids would act as endogenous mood stabilizers. Indeed, during episodes of depression levels of allopregnanolone were reported to be low [63], while the plasma concentration is elevated in patients with panic disorder [64], or after the induction of a panic attack [65]. Panic attack anxiety disorder is highly comorbid with type II bipolar disorder, and it has been suggested that the clinical manifestations of the panic attack is an expression of hyperthymia, specifically the “anxious hyperthymia”; this is a possible personality pre-morbid trait in panic disorder [71]. Both major depression and panic disorder are strongly bound to bipolar disorder, in terms of comorbidity [72], familiarity [73] and in a purported syndromic continuum with major depressive disturbance [74,75].

Irritable mood is another component of mood elevation in bipolar disorder. Anabolic/androgenic steroids increase sex drive and mental acuity. If abused, such steroids can cause irritability and impulsive aggression [76]. Social isolation in male mice and long-term treatment with anabolic steroids in female mice induces strong aggressive behavior towards intruders. In both sexes, a decrease of brain allopregnanolone is associated with such induced aggressive behavior [77]. Conversely, progesterone and its metabolite allopregnanolone have been implicated in suppressing irritability. Johannson et al. [78] conducted a study to determine whether or not a history of manic/hypomanic irritability is associated with low serum progesterone levels; they further tested whether single nucleotide polymorphisms (SNPs) in genes coding for steroidogenetic enzymes were coupled to previous manic irritability and/or with serum progesterone concentrations. They found that in males with bipolar disorders, progesterone concentrations were lower in those who had shown manic/hypomanic irritability compared with nonirritable patients. Specific SNPs were associated with manic/hypomanic irritability. Thus low progesterone levels and a cystine to serine change at position 145 in AKR1C4 gene were associated with manic/hypomanic irritability in males. Given that the enzyme AKR1C4 has both dehydrogenating and reductive activities in the steroidogenetic pathway, a missense variation in the gene may predispose to manic/hypomanic irritability by altering the relationship between progesterone and allopregnanolone.

More recently, the same group [79] found that in bipolar women, SNPs in AKR1C4 reduced the likelihood of exhibiting paranoid ideation during manic episodes by about 60%. Hence, gene variants in the steroidogenetic pathway and steroids concentration differences may be involved in the susceptibility to paranoia during mood elevation. Hardoy et al. [59] attempted to verify if differences in neurohormonal blood levels may be directly linked to some syndromal lifetime clusters (dimensions) using the Structural Clinical Interview for Mood Spectrum-Self Reported (SCI-MOODS-SR) [80] questionnaire of the mood spectrum independent of diagnosis in females with a lifetime diagnosis of major mood disorder (Bipolar Disorder, Major Depressive Disorder). This was done by investigating the patients during the luteal phase of their menstrual cycle and in a condition of clinical well-being. The analysis of the main components of the syndromal cluster evidenced the presence of 3 components identified by analysis of main components with Varimax rotation and Kaiser's normalization: 1) mania, 2) depression with mixed symptoms of agitation 3) irritable/elated cognition and suicidal ideas. Levels of allopregnanolone and progesterone were not associated with the mixed-depressive or purely manic syndromes, but rather with the symptom dimension characterized by irritable/elated cognition associated with suicidal thoughts. These results indicated that patients in euthymic, stabilized condition but with a history of irritable/elated symptoms mixed with suicide ideation had, at the evaluation time, higher blood levels of progesterone and derivates. These results are in apparent contradiction with the above discussed Johansson et al. [78] findings of low progesterone blood levels in bipolar patients of mixed states. However, taking into account the possibility that the steroids would act as endogenous mood stabilizers, this data can also be interpreted as patients with more severe mixed states need higher steroid levels to reach recovery compared to patients without past mixed symptoms. On the other hand, the hypothesis that steroids may be an endogenous mood stabilizer is supported by the evidence from case reports of recovery from post partum refractory mania [81]. Thus, progesterone and derivates may be particularly relevant on mixed – aggressive component of bipolar symtomathology.

It can also be hypothesized that a dysregulated system in patients with bipolar disorder would cause low levels of neuroactive steroids during depression and mixed states. Thus, drugs able to correct the malfunctioning of systems based on neurosteroids could improve clinical status in patients with bipolar disorder spectrum conditions.

Fluoxetine, which is effective in panic attack and major depression, also affect the levels of neurosteroids [66,67]. A role for neurosteroids in bipolar disorder is also consistent with the observation that a number of anticonvulsants (including valproate, lamotrigine and carbamazepine) are effective in bipolar disorder [82,83], an effect that could be partially due to action on neurosteroids. Therefore endogenous neuroactive steroids with anticonvulsant properties may play a role in the pathogenesis of bipolar disorders. Lithium is another effective therapeutic agent in bipolar disorder. Preclinical evidence suggests that lithium might induce its action via an effect on neurosteroids. The levels of allopregnanolone and pregnenolone were found significantly elevated in lithium-treated mice. Pregnenolone levels also tend to be higher following lithium treatment in humans [84].

Even the two atypical antipsychotics clozapine and olanzapine, effective against the manic phase of bipolar disorder [85,86], were proven to modify the levels of neurosteroids in animal studies. Clozapine markedly elevates pregnenolone in rat hippocampus, cerebral cortex, and serum; hippocampal levels were strongly correlated with serum levels. Olanzapine elevates pregnenolone levels, too, but to a lesser extent than clozapine [87,88]. Olanzapine, fluoxetine or their combination increased hippocampal pregnenolone and serum deoxycorticosterone in both higher- and lower-dose experiments, and elevated hippocampal allopregnanolone in higher-doses [89]. Since olanzapine and fluoxetine combination have clinical utility particularly in bipolar depression [90,91], and decrease of pregnenolone levels have been linked to depression, it is possible that olanzapine- and fluoxetine-induced pregnenolone elevations may contribute to the antidepressant actions of these agents in bipolar depression.

Effects of neurosteroids on mood fluctuation might extend across the bipolar disorder spectrum. Women of reproductive age with mental disorders may experience a fluctuating course of illness over the menstrual cycle. Some data suggest that for a subset of women there is a relationship between phases of the menstrual cycle and increased vulnerability for an exacerbation of ongoing mood disorders [22] (the so-called “catamenial mood disorder” [70]). A critical period is the one immediately following the birth of a child, when the level of progesterone neurosteroid derivatives drop; the post-natal period is linked to an increased vulnerability to the onset or recurrence of mood disorders [92].

### Neuroactive steroids and GABAergic drugs in bipolar disorders

Recent research data from some GABAergic compounds, including gabapentin [93,94], tiagabine [95], topiramate [96], have produced disappointing and conflicting results as far as their effectiveness in bipolar disorder is concerned. Preliminary evidence of their effectiveness in patients diagnosed with bipolar disorder was not confirmed by subsequent randomized, placebo-controlled studies [97-99].

Most antiepileptic compounds exert a direct or indirect GABA-mediated inhibitory action [100]. However, the impact of the neuroactive steroids on the structure of the GABA_A_ receptor is a factor that has not been adequately examined in the investigation of the pharmacological action of putatively GABAergic drugs. Indeed, patients with bipolar disorder often abuse alcohol or drugs such as benzodiazepines [101], which can induce changes in the heteropentameric structure of the GABA_A_ receptor, changes that may alter the subsequent action of drugs targeting the GABA_A_ receptor. In addition, benzodiazepines such as diazepam or midazolam were found to promote neurosteroid synthesis [102-104]. Moreover, if neurosteroids really are involved in the etiology of bipolar disorder and their levels fluctuate during the different phases of the disorder, the influence of neurosteroids on the GABA_A_ receptors also will fluctuate. This will further modify the responsiveness to GABAergic compounds. Effectiveness of potentially antimanic GABAergic drugs would depend of the status of the GABA_A_ receptors on which they exert their action, and on levels of neurosteroids acting on the GABA_A_ receptors. Overall, the chance of finding a therapeutic effectiveness of GABAergic compounds will be a function of: a) inter-individual differences in neurosteroids synthesis, secretion and action at the target receptor; b) the conformation of the GABA_A_ receptor as a function of alcohol and/or preceding treatment with drugs acting on it; the phase of the disorder, whether depressive or excitatory. Gender differences in neurosteroids functioning also would have an impact on the effectiveness of GABAergic compounds in bipolar disorder, and should be accounted for.

## Conclusions

Literature on the role of neuroactive steroids in mental disorders is sparse. Nonetheless we have made an attempt to present a narrative review of existing studies on neurosteroids acting on the GABAergic receptors. Current evidence suggests that the investigation of neuroactive steroids on mental disorders might open new frontiers in the investigation of the etiology and treatment of mood disorders, particularly bipolar disorder. Neurosteroids might be an endogenous mood stabilizer and the alteration of their functioning on a genetic or biochemical level might be responsible for the display of symptoms in individuals vulnerable to bipolar disorder.

## Competing interests

The authors’ declare that they have no competing interest.

## Authors’ contributions

MGC conceived the idea of the paper and drafted the manuscript after discussion with KMB and AP. KMB and AP contributed to the molecular (particularly KMB) and clinical (AP) aspects of the paper. All authors read and approved the final manuscript.
